# In Vitro Glucose Measurement from NIR and MIR Spectroscopy: Comprehensive Benchmark of Machine Learning and Filtering Chemometrics

**DOI:** 10.1016/j.heliyon.2024.e30981

**Published:** 2024-05-09

**Authors:** Heydar Khadem, Hoda Nemat, Jackie Elliott, Mohammed Benaissa

**Affiliations:** a Department of Electronic and Electrical Engineering, University of Sheffield, UK; b Department of Computer Science, University of Manchester, Manchester, UK; c Artificial Intelligence & Machine Learning Team, KultraLab, London, UK; d Department of Oncology and Metabolism, University of Sheffield, UK; e Sheffield Teaching Hospitals, Diabetes and Endocrine Centre, Northern General Hospital, Sheffield, UK

**Keywords:** Near-infrared, Mid-infrared, Spectroscopy, Machine learning, Artificial intelligence, Glucose, Signal processing

## Abstract

The quantitative analysis of glucose using spectroscopy is a topic of great significance and interest in science and industry. One conundrum in this area is deploying appropriate preprocessing and regression tools. To contribute to addressing this challenge, in this study, we conducted a comprehensive and novel comparative analysis of various machine learning and preprocessing filtering techniques applied to near-infrared, mid-infrared, and a combination of near-infrared and mid-infrared spectroscopy for glucose assay. Our objective was to evaluate the effectiveness of these techniques in accurately predicting glucose levels and to determine which approach was most optimal. Our investigation involved the acquisition of spectral data from samples of glucose solutions using the three aforementioned spectroscopy techniques. The data was subjected to several preprocessing filtering methods, including convolutional moving average, Savitzky-Golay, multiplicative scatter correction, and normalisation. We then applied representative machine learning algorithms from three categories: linear modelling, traditional nonlinear modelling, and artificial neural networks. The evaluation results revealed that linear models exhibited higher predictive accuracy than nonlinear models, whereas artificial neural network models demonstrated comparable performance. Additionally, the comparative analysis of various filtering methods demonstrated that the convolutional moving average and Savitzky-Golay filters yielded the most precise outcomes overall. In conclusion, our study provides valuable insights into the efficacy of different machine learning techniques for glucose measurement and highlights the importance of applying appropriate filtering methods in enhancing predictive accuracy. These findings have important implications for the development of new and improved glucose quantification technologies.

## 1 Introduction

Glucose monitoring is an example of a promising area of research with a wide variety of applications in different fields. With the help of machine learning, it has become promising to accurately predict glucose levels using methods such as near-infrared (NIR) and mid-infrared (MIR) spectroscopy. These techniques involve analysing the interaction of light with the investigated substance to obtain information about glucose levels [[1], [2], [3], [4], [5], [6], [7], [8], [9], [10], [11], [12], [13], [14], [15], [16]].

A variety of modalities have been explored for glucose monitoring, including optical, electrical, and acoustic methods. Optical methods, such as NIR and MIR spectroscopy, have received significant attention due to their ability to measure glucose levels effectively by analysing the absorption spectra of glucose molecules. Other methods include Raman spectroscopy, optical coherence tomography, and photoacoustic spectroscopy, which have shown some promise for glucose identification but require further investigation to determine their comprehensive utility. Despite the ongoing research efforts, there is no single modality that has emerged as the gold standard for glucose quantification. However, NIR and MIR spectroscopy are among the most promising modalities, as they have shown comparatively high accuracy and reliability. Therefore, further research is needed to explore and optimise these modalities for practical [[17], [18], [19], [20]].

Over the years, various machine learning approaches have been utilized for glucose quantification using NIR and MIR spectroscopy. These approaches can be broadly categorised into classical linear methods such as partial least squares regression (PLSR) and principal component regression (PCR), classical nonlinear methods such as support vector regression (SVR) and random forest regression (RFR), and neural network methods such as multilayer perceptron (MLP) and long short-term memory (LSTM). These methods have shown promising results in accurately predicting glucose concentrations from spectroscopic data. However, each method has its own strengths and limitations, and the selection of an appropriate method largely depends on the characteristics of the data and the specific application. Further research is needed to optimise and validate these machine learning approaches for glucose monitoring [[21], [22], [23], [24], [25], [26], [27], [28]].

In glucose assay using NIR and MIR spectroscopy, preprocessing filtering methods are applied to the acquired spectra to enhance the signal-to-noise ratio and remove unwanted spectral variations caused by different factors. Several filtering methods have been proposed for this purpose, such as convolutional moving average (CMA), Savitzky-Golay (SG), and multiplicative scatter correction (MSC). These preprocessing filtering methods are essential for improving the accuracy and reliability of machine learning models in glucose identification [[29], [30], [31], [32]].

Despite significant advances in spectroscopic glucose quantification, a comprehensive and comparative analysis of preprocessing and regression techniques remains lacking. This study addresses this gap by undertaking a detailed evaluation of various machine learning strategies applied to the quantitative analysis of glucose using NIR, MIR, and combined NIR-MIR spectroscopy. By exploring the influence of different preprocessing filters on the effectiveness of these models, this research provides a systematic assessment of their impact on model performance.

Our findings are particularly crucial for researchers and healthcare professionals focused on enhancing glucose measurement techniques. This study not only contributes to the existing body of knowledge but also lays a solid foundation for future research in this critical area. The main contributions of our work are:

• An extensive comparative assessment of various machine learning techniques applied to glucose detection across NIR, MIR, and combined NIR-MIR spectroscopic methods.
• An analysis of how different preprocessing filters affect the efficacy of machine learning algorithms in spectroscopic glucose quantification.
• Insights on the enhanced accuracy achieved through the integration of NIR and MIR spectroscopy for glucose measurement.
• Strategic guidance on selecting preprocessing filters to improve the precision of machine learning predictions in glucose assays.

Overall, this study can aid in the development of accurate and reliable glucose monitoring techniques. The remainder of the article is organised as follows: Section 2 describes the data collection process and the dataset used. Section 3 details the comparative analysis framework designed for this work. Section 4 presents the results achieved. Section 5 includes relevant discussions related to the results obtained. Section 6 summarises the study and concludes the paper, while Section 7 presents several avenues for future exploration.

## 2 Material

In this section, first, we explain spectroscopic modalities used for glucose sensing. Afterwards, we describe the experimental data used for the analysis and spectra collection process.

### 2.1 Spectroscopic modalities

#### 2.1.1 NIR spectra

NIR spectroscopy is a non-destructive analytical technique that uses light in the NIR region to identify and quantify the chemical and physical properties of a sample. In glucose identification, NIR spectroscopy has been widely used as a fast and non-invasive method for measuring glucose levels in biological samples such as blood, plasma, and saliva. NIR spectroscopy works by measuring the absorbance of NIR light by chemical bonds in the sample, which are related to the concentration of glucose in the sample [[33], [34], [35]].

#### 2.1.2 MIR spectra

MIR spectroscopy is another analytical technique that uses light in the MIR region to identify and quantify the chemical and physical properties of a sample. In glucose assay, MIR spectroscopy has also been used to measure glucose levels in biological samples. MIR spectroscopy works by measuring the vibrational modes of chemical bonds in the sample, which are also related to the concentration of glucose in the sample [[36], [37], [38], [39]].

#### 2.1.3 NIR-MIR spectra

NIR-MIR spectroscopy combines the strengths of both NIR and MIR spectroscopy by measuring the absorbance of light in both the NIR and MIR regions. This allows for a more comprehensive analysis of the sample, as different types of chemical bonds can be measured in each region. In glucose identification, NIR-MIR spectroscopy has been shown to improve the accuracy of glucose measurement compared to using NIR or MIR spectroscopy alone. This is because glucose has characteristic absorbance bands in both the NIR and MIR regions, which can be used to improve the accuracy and sensitivity of glucose measurement [[40], [41], [42], [43]].

### 2.2 Experimental data

#### 2.2.1 Sample preparation

The experimental samples were prepared at the University of Sheffield's Department of Chemistry. Two separate 0.5-L aqueous solutions with the same pH level (7.4), phosphate concentration (0.01 M/dl), and human serum albumin concentration (5 g/dl) were created. One of the solutions contained 500 mg/dL glucose, while the other did not. The first sample, containing 500 mg/dL glucose, was formed by preserving 5 mL of the first solution. Subsequently, 5 mL of the second solution was added to the first solution, lowering the glucose concentration to 495 mg/dL, and forming the second sample. The process was repeated until 100 samples with glucose concentrations ranging from 5 to 500 mg/dL in 5 mg/dL increments were acquired [28,31].

#### 2.2.2 Spectra acquisition

**Fig. 1:**
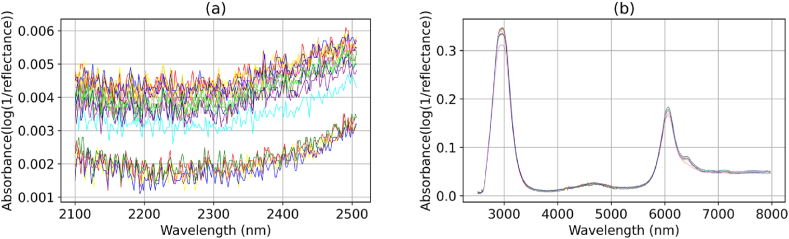
Twenty raw spectra were randomly chosen from chemical samples, consisting of signals in the (**a**) near-infrared and (**b**) mid-infrared regions.

In uncontrolled laboratory conditions, spectra were collected using a Fourier transform infrared spectrometer (PerkinElmer Inc., USA) at the University of Sheffield's Department of Materials Science and Engineering. The sensing lens of the device was wiped clean with ethanol before each sample was placed for recording. Then, a layer of the sample was overlaid onto the entire surface of the lens, and the spectrometer recorded the absorption signals using the attenuated total reflection technique. The recorded spectra ranged in wavelength from 2100 to 8000 nm with a resolution of 1.7 nm. The wavelengths within 2100–2500 nm and 2500–8000 nm belonged to the NIR and MIR regions, respectively. To ensure accuracy, the spectrometer was configured to take four readings for each sample and return the average as the output [28,31]. Fig. 1 displays some of the collected raw spectra.

## 3 Methods

**Fig. 2:**
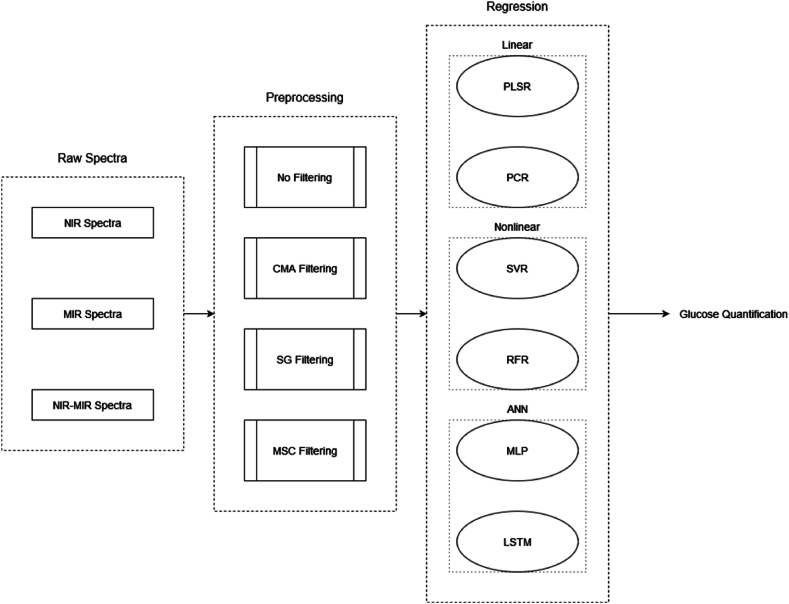
Block diagram of the comparative analysis of different filtering and regression approaches considered for quantitative analysis of glucose from NIR, MIR, and NIR-MIR spectra. Note. ANN: Artificial Neural Networks; LSTM: Long Short-Term Memory; CMA: Convolutional Moving Average; MSC: Multiplicative scatter Correction; NIR: Near-infrared; PCR: Principal Component Regression; PLSR: Partial Least Square Regression; RFR: Random Forest Regression; SG: Savitzky-Golay; SVR: Support Vector Regression; MLP: Multilayer Perceptron.

This section initially discusses filtering approaches considered in this work for comparative benchmark investigations. After that, we detail representative machine learning techniques assigned for our quantitative analysis of glucose. Finally, we represent the evaluation criteria used for our side-by-side analysis. The block diagram in Fig. 2 depicts the workflow considered for our comparative analysis.

### 3.1 Spectra filtering techniques

In this study, we chose CMA, SG, and MSC as they collectively represent a broad range of filtering techniques commonly used in spectroscopic analysis.

#### 3.1.1 CMA filter

CMA is a simple smoothing technique used in spectroscopic data analysis. It works by taking the average of a sliding window of adjacent data points. The size of the window determines the level of smoothing, with larger windows resulting in more smoothing. CMA is easy to apply and can effectively reduce noise in spectral data. However, it has limitations in preserving spectral features, especially in the presence of sharp peaks and rapid changes in the spectra [44,45]. In this study, the size of the window is 7. CMA was selected for its simplicity and effectiveness in smoothing data by averaging, which is representative of moving average techniques.

#### 3.1.2 SG filter

SG filtering is a popular preprocessing technique used in spectroscopic data analysis. It is a type of polynomial smoothing that fits a series of consecutive data points with a polynomial function, which is then used to estimate the value of each data point. Savitzky-Golay filtering is effective in reducing noise while preserving spectral features, such as sharp peaks and baseline curvature. It is also computationally efficient and can handle data with irregular spacing. SG filtering is widely used in many spectroscopic applications, including Raman and infrared spectroscopy [[46], [47], [48], [49]]. In this study, we applied the SG filter with a window size of 5, a polynomial order of 3, and a zero-order derivative. SG was included due to its ability to preserve high-frequency spectral features while smoothing, a quality that makes it exemplary of polynomial regression-based smoothing methods.

#### 3.1.3 MSC filter

MSC is a preprocessing technique used in spectroscopic data analysis to correct for unwanted variations in the spectra caused by sample-to-sample differences in scattering, path length, and other factors. MSC works by using a reference spectrum that represents the average of all the spectra in the dataset and then scaling each spectrum to remove the influence of unwanted variation. MSC is effective in removing spectral variations that are not related to the analyte of interest, which can improve the accuracy and reliability of the spectral analysis [50]. In this study, we took the average of all signals in the calibration set to serve as our reference spectrum. We then adjusted each individual signal with respect to this reference to ensure uniform scatter levels across all signals. MSC was included in our analysis since it is a standard choice for correcting scatter effects.

### 3.2 Machine learning regression models

#### 3.2.1 Linear models

• PLSR is a machine learning method used for modelling relationships between two sets of variables, where one set of variables is the predictor and the other set is the response. PLSR has shown great potential in spectroscopic data analysis, where it is used to build quantitative models for predicting properties of interest based on spectral data. PLSR can handle highly collinear and noisy spectral data, and it can identify spectral regions that are most informative for predicting the property of interest. has become a popular tool in spectroscopic analysis and has been successfully applied in various fields, including food science, environmental analysis, and pharmaceuticals [51,52].
• PCR is a machine learning technique used to analyse spectroscopic data. It involves reducing the dimensionality of the data by transforming the original variables into a smaller set of principal components. These principal components represent linear combinations of the original variables that capture the maximum variance in the data. PCR then uses these principal components as predictors in a regression model to predict the outcome variable of interest. PCR has potential advantages for spectroscopic data analysis, as it can handle high-dimensional data with many correlated variables. It also allows for the identification of important spectral features that contribute to the prediction of the outcome variable. Overall, PCR is a powerful tool for analysing spectroscopic data and has many potential applications in various fields [[53], [54], [55]].

#### 3.2.2 Traditional nonlinear machine learning

• SVR is a machine learning algorithm that is widely used for predicting continuous outcomes from spectroscopic data. In SVR, the data are transformed into a high-dimensional feature space where the algorithm finds the optimal hyperplane that maximally separates the data into different classes. SVR can handle non-linear relationships between the input features and the outcome variable by using kernel functions to map the input features into a higher-dimensional space. SVR has been successfully applied in many spectroscopic studies, such as in the analysis of complex mixtures, where it outperforms traditional linear regression methods. Its ability to handle complex data makes it a powerful tool for spectroscopic data analysis, particularly in fields such as spectroscopy and bioinformatics [56,57].
• RFR is a popular machine learning algorithm used for predictive modelling. It is a type of ensemble learning method that creates multiple decision trees and combines them to make a final prediction. The algorithm works by randomly selecting a subset of features and data points from the dataset, and then constructing a decision tree for each subset. Each tree is trained independently and their predictions are averaged to make the final prediction. RFR is highly effective for spectroscopic data analysis because it can handle high-dimensional data with complex interactions and non-linear relationships between variables [[58], [59], [60], [61], [62]].

#### 3.2.3 Artificial neural network

• MLP is a type of artificial neural network commonly used in machine learning for regression and classification tasks. MLP consists of multiple layers of nodes, where each node represents an artificial neuron that applies a nonlinear activation function to a weighted sum of its inputs. MLP is particularly powerful for spectroscopic data analysis because it can automatically learn complex patterns in high-dimensional data and capture nonlinear relationships between variables. MLP can also handle noisy signals by using regularization techniques [63,64].
• LSTM is a type of recurrent neural network that is designed to capture temporal dependencies in sequential data. LSTM consists of memory cells that can selectively retain or forget information from previous time steps, allowing it to model long-term dependencies and handle variable-length sequences. LSTM is particularly powerful for spectroscopic data analysis because it can capture complex temporal patterns, such as the evolution of spectral features [[65], [66], [67]].

### 3.3 Models configuration

This section presents detailed outcomes of the hyperparameter tuning process for all machine learning models discussed in the paper. We employed a grid search methodology to systematically explore and identify optimal settings for each model's hyperparameters, aiming to maximise performance on the training dataset.

A grid search involves defining a comprehensive search space for each hyperparameter and evaluating model performance for each combination. This method ensures that we identify the combination of parameters that yields the best performance, measured in terms of prediction accuracy, overfitting control, and computational efficiency.

The search spaces for each model's hyperparameters were selected based on a combination of literature review, preliminary empirical tests, and standard practices within the field. The rationale for choosing specific ranges is to balance the model's complexity with its ability to generalise to new data. Below are the hyperparameter tuning details for each model. The chosen values for each hyperparameter, indicated with an asterisk, were those that provided the best balance between accuracy and generalisation as evidenced in the training phase. These selections are directly reflected in the performance outcomes detailed in the main results section.

#### 3.3.1 Linear models

• PLSR:
  Number of components: {2, 4, 6*, 8, 10}
    ⁃ Purpose: To evaluate the incremental benefit of adding more components against the risk of overfitting.
  Scaling: {True, False*}
    ⁃ Purpose: To investigate the impact of feature scaling on model performance.
  Max iteration: {500, 1000, 1500*, 2000, 2500}
    ⁃ Purpose: Set to ensure convergence of the algorithm under different complexity scenarios.
• PCR:
  Number of components: {2, 4, 6, 8*, 10}
    ⁃ Purpose: To optimise the number of principal components that capture the most variance without introducing noise.
  SVD solver: {Auto, Full, Arpack, Randomised*}
    ⁃ Purpose: Varied to find the most efficient computational approach for decomposing the data matrix.
  Intercept term: {True, False*}
    ⁃ Purpose: Assessed to establish whether including an intercept improves model predictions.

#### 3.3.2 Nonlinear models

• SVR:
  Penalty term: {0.1, 1*, 10, 100, 1000}
    ⁃ Purpose: To adjust the regularisation parameter which balances the trade-off between achieving a low error on the training data and minimising model complexity for better generalisation.
  Gamma: {scale, auto, 0.1, 1, 10*}
    ⁃ Purpose: Controls the curvature of the decision boundary; varied to adapt the model to the data distribution.
  Kernel: {poly*, rbf, sigmoid}
    ⁃ Purpose: To select the type of hyperplane used to separate the data, offering different ways of handling non-linear data.
• RFR:
  Number of estimators: {50, 100, 200, 300, 400*}
    ⁃ Purpose: To determine the optimal number of trees that contribute to an accurate prediction without overfitting.
  Max depth: {5, 10*, 15, 20, 25}
    ⁃ Purpose: Controls the depth of each tree; deeper trees capture more detailed data specifics but risk overfitting.
  Min sample split: {2, 5*, 10, 15, 20}
    ⁃ Purpose: The minimum number of samples required to split an internal node; affects the tree depth and complexity.

#### 3.3.3 ANN models

• MLP:
  Number of nodes: {16*, 32, 64, 128, 256}
    ⁃ Purpose: To determine the optimal size of the network's layers to efficiently process features without overfitting. More nodes can capture complex patterns but may increase the risk of memorising the training data.
  Activation functions: {identity, logistic, tanh, relu*}
    ⁃ Purpose: To test different types of nonlinear transformations to find which best facilitates the learning process in hidden layers. Each function offers different benefits, such as improving gradient flow or introducing non-linearity.
  Max iteration: {500, 1000*, 1500, 2000, 2500}
    ⁃ Purpose: To ensure sufficient training epochs for the network to converge to a solution, balancing between adequate training time and prevention of overfitting.
• LSTM:
  LSTM units: {16, 32*, 64, 128, 256}
    ⁃ Purpose: To optimise the number of units in LSTM layers, which affects the model's ability to capture long-term dependencies in time-series data. More units can enhance learning capacity at the cost of computational efficiency.
  Dense units: {16, 32*, 64, 128, 256}
    ⁃ Purpose: To adjust the number of neurons in the dense layers following the LSTM layers, which helps in forming connections and improving learning from the LSTM outputs.
  Activation functions: {identity, logistic, tanh, relu*}
    ⁃ Purpose: Similar to MLP, to identify the optimal activation function that helps the LSTM layers and dense layers effectively capture nonlinear relationships in the data.

### 3.4 Evaluation criteria

#### 3.4.1 MAE metric

MAE measures the average magnitude of the errors in a set of predictions, without considering their direction. It is calculated by taking the absolute value of the difference between the predicted values and actual values and then averaging these absolute differences over the dataset (Eq. (1)).

(1) $$MAE=\left(\sum_{i=1}^{N}\left|{BGL}_{i}-\;{B\hat{G}L}_{i}\right|\right)/N$$

• ${BGL}_{i}$: the actual blood glucose level in the ith test instance.
• ${B\hat{G}L}_{i}$: the predicted blood glucose level for the ith test instance.
• *N:* is the total number of observations in the test set.

MAE is a simple and easy-to-interpret measure of the overall accuracy of a regression model. A smaller MAE value indicates that the model has better accuracy in predicting the outcome variable. In spectroscopic data analysis, MAE is commonly used to evaluate the performance of regression models in predicting the concentration of a specific component in a sample. By comparing the MAE values of different models, researchers can select the most accurate one for their analysis [68,69].

#### 3.4.2 RMSE metric

RMSE is a quadratic scoring rule that measures the average magnitude of the error. It's the square root of the average of squared differences between prediction and actual observation. It is calculated as follows (Eq. (2)).

(2) $$RMSE=\sqrt{\left(\sum_{i=1}^{N}{\left({BGL}_{i}-\;{B\hat{G}L}_{i}\right)}^{2}\right)/N}$$

• ${BGL}_{i}$: the actual blood glucose level in the ith test instance.
• ${B\hat{G}L}_{i}$: the predicted blood glucose level for the ith test instance.
•*N:* is the total number of observations in the test set.

A smaller RMSE indicates a better fit between the model and the data. It is commonly used in scientific studies to assess the accuracy of model predictions [70].

#### 3.4.3 MAPE metric

MAPE is a measure of the percentage difference between predicted and observed values. It calculates the absolute percentage difference between predicted and observed values and then averages it across all observations. MAPE is calculated as Eq. (3).

(3) $$MAPE=\left(\left(\sum_{i=1}^{N}\left|\left({BGL}_{i}-\;{B\hat{G}L}_{i}\right)/{BGL}_{i}\right|\right)/N\right)\times 100$$

• ${BGL}_{i}$: the actual blood glucose level in the ith test instance.
• ${B\hat{G}L}_{i}$: the predicted blood glucose level for the ith test instance.
•*N:* is the total number of observations in the test set.

It provides an understanding of the relative size of the error in comparison to the actual value. MAPE is specifically useful when comparing models with different units or scales of measurement [71,72].

#### 3.4.4 Coefficient of Determination (R^2^)

R^2^ (Eq. (4)) is a measure of the proportion of variability in the response variable that is explained by the regression model. It ranges from 0 to 1, with higher values indicating a better fit. R^2^ is commonly used in scientific studies to assess the strength of the relationship between the predictor variables and the response variable [[73], [74], [75]].

(4) $$R^{2}=(1-(\left(\sum_{i=1}^{N}{\left({BGL}_{i}-\;{B\hat{G}L}_{i}\right)}^{2}\right)/\left(\sum_{i=1}^{N}{\left({BGL}_{i}-\bar{BGL}\right)}^{2}\right)))\times 100\;$$

• ${BGL}_{i}$: the actual blood glucose level in the ith test instance.
• ${B\hat{G}L}_{i}$: the predicted blood glucose level for the ith test instance.
•*N:* the total number of observations in the test set.
• $\bar{BGL}$: the mean of actual blood glucose levels over the N observations.

### 3.5 Data partitioning

For the development of our quantitative models, we utilised stratified sampling to partition the dataset into training and testing subsets. This method ensured that each subset was representative of the overall dataset, maintaining similar distributions of glucose concentrations. Specifically, 80 % of the data points were randomly selected within each stratum to form the training set, which was used for both model training and hyperparameter tuning. The remaining 20 % of the data points, again selected randomly within each stratum, constituted the testing set, reserved exclusively for model evaluation. This approach ensures that the testing data remains unseen during the model development phase, thereby preventing data leakage and ensuring a robust evaluation of model performance. The mean glucose concentration in the training set was 250.3 mg/dL with a standard deviation of 146.4 mg/dL, while the testing set had a mean concentration of 261.21 mg/dL with a standard deviation of 35.2 mg/dL.

### 3.6 Comparison analysis

In this study, we performed a systematic ranking analysis to evaluate and compare the performance of various machine learning models and preprocessing filters for glucose quantification using NIR, MIR, and combined NIR-MIR spectroscopy. This subsection outlines the methodology employed in the ranking process.

Models and filters were ranked separately for each spectroscopy technique—NIR, MIR, and combined NIR-MIR. Rankings for each performance metric—MAE, RMSE, MAPE, and R^2^—were assigned from the best performing (rank 1) to the least performing. Lower values in MAE, RMSE, and MAPE indicated superior performance, earning a model or filter a higher rank. Conversely, a higher R^2^ value resulted in a higher rank, reflecting a better fit between model predictions and actual measurements.

To provide an overarching assessment of performance, an aggregate ranking for each model and filter was calculated. This was determined by averaging the ranks obtained for each metric, allowing for a comprehensive comparison across all considered aspects of performance.

The outcomes of the ranking analysis are visualised in figures within the Results section. These visual aids facilitate a clear and direct comparison of the performance of models and filters across different parameters, highlighting which models and filters consistently perform well across various evaluation metrics.

## 4 Results

This section represents the evaluation results for glucose quantification models generated from NIR, MIR, and NIR-MIR signals.

### 4.1 NIR region

The results of the study for the NIR region are presented in Table 1. The linear models, PLSR and PCR, outperformed the nonlinear models, SVR and RFR, in terms of predictive ability. The ANN models, MLP and LSTM, performed similarly to the linear models.

• The PLSR and PCR algorithms showed moderate predictive ability with MAE ranging from 65.1 to 68.5 mg/dL, RMSE ranging from 96.0 to 99.5 mg/dL, and MAPE ranging from 62.4 % to 69.4 %. The R^2^ values ranged from 44.5 % to 50.7 %.
• The ANN models demonstrated similar performance, with MAE ranging from 66.8 to 75.4 mg/dL, RMSE ranging from 97.2 to 100.1 mg/dL, and MAPE ranging from 65.4 % to 71.4 %. The R^2^ values ranged from 43.2 % to 51.9 %.
• In contrast, the SVR and RFR algorithms exhibited poorer predictive ability, with MAE ranging from 68.2 to 76.4 mg/dL, RMSE ranging from 99.1 to 102.4 mg/dL, and MAPE ranging from 64.2 % to 72.8 %. The R^2^ values ranged from 42.0 % to 47.4 %.

**Table 1:** Results of evaluation analysis for glucose quantification models created in the NIR region.

Method	Model	Filter	MAE (mg/dL)	RMSE (mg/dL)	MAPE (%)	R^2^ (%)
Linear	PLSR	None	67.7	98.1	66.6	47.1
		CMA	65.3	96.2	62.4	51.8
		SG	65.1	96.0	62.5	51.6
		MSC	71.2	104.8	70.1	44.5
	PCR	None	68.1	99.5	69.4	46.5
		CMA	68.2	99.4	67.1	46.4
		SG	66.7	97.8	64.5	50.7
		MSC	68.5	99.8	70.1	46.2
Nonlinear	SVR	None	76.2	102.4	72.8	42.0
		CMA	76.4	101.7	71.3	42.3
		SG	76.3	101.6	71.0	42.2
		MSC	76.2	101.4	71.1	42.8
	RFR	None	70.1	99.4	65.1	46.9
		CMA	72.4	102.3	67.9	45.2
		SG	72.5	102.1	68.8	44.9
		MSC	68.2	99.1	64.2	47.4
ANN	MLP	None	67.1	97.8	65.9	48.1
		CMA	66.9	97.2	65.4	51.2
		SG	66.8	97.3	65.2	51.9
		MSC	74.5	105.1	69.4	45.1
	LSTM	None	75.4	100.1	69.9	43.4
		CMA	74.2	99.7	71.4	43.6
		SG	74.3	99.3	69.0	43.2
		MSC	74.1	99.4	70.1	44.8

Note. ANN: Artificial Neural Networks; LSTM: Long Short-Term Memory; CMA: Convolutional Moving Average; MAE: Mean Absolute Error; MAPE: Mean Absolute Percentage Error; MSC: Multiplicative scatter Correction; NIR: Near-infrared; PCR: Principal Component Regression; PLSR: Partial Least Square Regression; R^2^: Coefficient of Determination; RFR: Random Forest Regression; RMSE: Root Mean Square Error; SG: Savitzky-Golay; SVR: Support Vector Regression; MLP: Multilayer Perceptron.

The evaluation results also allowed for the comparison of the performance of different filters. The CMA and SG filters outperformed the None and MSC filters across all evaluation metrics.

**Fig. 3:**
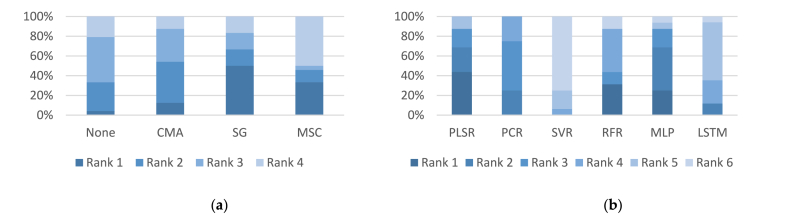
Results of comparative analysis in the NIR region between different: (**a**) filtering models; (**b**) regression models. Note. ANN: Artificial Neural Networks; LSTM: Long Short-Term Memory; CMA: Convolutional Moving Average; MSC: Multivariate Scatter Correction; MLP: Multilayer Perceptron; NIR: Near-infrared; PCR: Principal Component Regression; PLSR: Partial Least Square Regression; RFR: Random Forest Regression; SG: Savitzky-Golay; SVR: Support Vector Regression.

Fig. 3 depicts the performance rankings of the NIR models across various filtering and regression techniques, highlighting that SG and MSC ranked highest among the filtering techniques while PLSR and RFR recorded the best rankings overall among regression methods.

### 4.2 MIR region

The results for the MIR region are detailed in Table 2. Linear models, including PLSR and PCR, varied in performance based on the applied filter:

• PLSR showed MAE values ranging from 65.1 to 71.2 mg/dL, RMSE values from 96.0 to 104.8 mg/dL, MAPE values from 62.4 % to 70.1 %, and R^2^ values from 44.5 % to 51.8 %.
• PCR Demonstrated MAE values from 66.7 to 68.5 mg/dL, RMSE from 97.8 to 99.5 mg/dL, MAPE from 64.5 % to 69.4 %, and R^2^ from 46.2 % to 50.7 %.

**Table 2:** Results of evaluation analysis for glucose quantification models created in the MIR region.

Method	Model	Filter	MAE (mg/dL)	RMSE (mg/dL)	MAPE (%)	R^2^ (%)
Linear	PLSR	None	25.7	36.2	28.0	92.0
		CMA	24.3	35.2	27.8	92.8
		SG	24.6	36.1	28.1	91.8
		MSC	26.1	38.2	29.1	89.7
	PCR	None	25.1	35.8	27.4	92.1
		CMA	24.2	34.9	27.9	92.4
		SG	25.2	35.4	28.1	91.2
		MSC	28.9	39.5	32.1	88.1
Nonlinear	SVR	None	25.2	34.9	27.1	92.4
		CMA	26.1	35.8	27.6	90.8
		SG	26.2	35.7	27.5	90.6
		MSC	26.4	35.6	29.1	89.2
	RFR	None	25.1	36.1	27.9	92.1
		CMA	32.4	43.1	34.1	79.8
		SG	33.1	43.2	33.8	79.4
		MSC	32.2	41.2	33.2	80.1
ANN	MLP	None	25.1	35.8	27.4	91.1
		CMA	24.1	34.2	26.9	92.3
		SG	24.2	34.1	26.8	92.1
		MSC	28.1	39.2	29.3	87.1
	LSTM	None	24.1	35.8	27.1	93.1
		CMA	24.3	36.1	26.9	92.4
		SG	24.5	36.2	26.4	92.3
		MSC	23.9	36.1	27.4	92.5

Note. ANN: Artificial Neural Networks; LSTM: Long Short-Term Memory; CMA: Convolutional Moving Average; MAE: Mean Absolute Error; MAPE: Mean Absolute Percentage Error; MIR: Mid-infrared; MLP: Multilayer Perceptron; MSC: Multiplicative scatter Correction; PCR: Principal Component Regression; PLSR: Partial Least Square Regression; R^2^: Coefficient of Determination; RFR: Random Forest Regression; RMSE: Root Mean Square Error; SG: Savitzky-Golay; SVR: Support Vector Regression.

Nonlinear models, including SVR and RFR, showed poorer predictive ability:

• SVR exhibited MAE values from 68.2 to 76.4 mg/dL, RMSE from 99.1 to 102.4 mg/dL, MAPE from 64.2 % to 72.8 %, and R^2^ from 42.0 % to 47.4 %.
• RFR recorded MAE from 68.2 to 72.5 mg/dL, RMSE from 99.4 to 102.3 mg/dL, MAPE from 64.2 % to 68.8 %, and R^2^ from 44.9 % to 47.4 %.

ANN models, including MLP and LSTM, showed moderate predictive ability:

• MLP had MAE values from 66.8 to 75.4 mg/dL, RMSE from 97.2 to 100.1 mg/dL, MAPE from 65.4 % to 71.4 %, and R^2^ from 43.2 % to 51.9 %.
• LSTM showed MAE from 74.1 to 75.4 mg/dL, RMSE from 99.4 to 100.1 mg/dL, MAPE from 69.0 % to 71.4 %, and R^2^ from 43.2 % to 44.8 %.

**Fig. 4:**
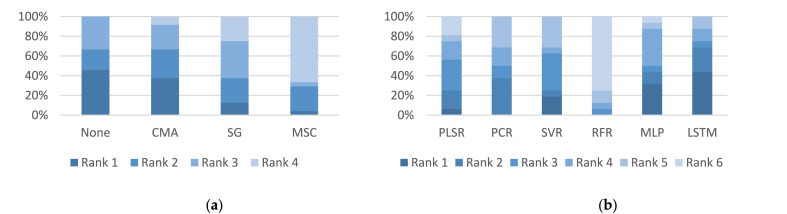
Results of comparative analysis in the MIR region between different: (a) filtering models; (b) regression models. Note. ANN: Artificial Neural Networks; LSTM: Long Short-Term Memory; MIR: Mid-infrared; CMA: Convolutional Moving Average; MLP: Multilayer Perceptron; MSC: Multivariate Scatter Correction; PCR: Principal Component Regression; PLSR: Partial Least Square Regression; RFR: Random Forest Regression; SG: Savitzky-Golay; SVR: Support Vector Regression.

Additionally, Fig. 4 provides a visual comparison of the performance rankings of the MIR models across different filtering and regression techniques, highlighting the standout performance of no-filtering and CMA methods in filtering, and LSTM and MLP in regression approaches.

### 4.3 NIR-MIR region

The study's results for the NIR-MIR region, detailed in Table 3, demonstrate that the linear models, PLSR and PCR, generally performed better than the nonlinear models, SVR and RFR, and the ANN models, MLP and LSTM, in terms of predictive accuracy:

• PLSR and PCR models showed superior predictive ability with MAE ranging from 25.2 to 28.3 mg/dL, RMSE from 35.3 to 39.4 mg/dL, and MAPE from 28.2 % to 29.3 %. The R2 values for these models ranged from 89.3 % to 93.1 %.
• SVR and RFR models exhibited similar predictive accuracy with MAE for SVR from 25.1 to 25.5 mg/dL, RMSE from 34.8 to 35.4 mg/dL, and MAPE from 28.0 % to 28.5 %. The R2 values ranged from 92.7 % to 93.8 %.
• MLP and LSTM models performed comparably to linear models, with MAE from 24.0 to 27.7 mg/dL, RMSE from 34.0 to 39.9 mg/dL, and MAPE from 26.5 % to 29.5 %. The R2 values ranged from 87.9 % to 93.3 %.
• The RFR algorithm displayed poorer predictive accuracy, particularly with MAE ranging from 25.4 to 33.2 mg/dL, RMSE from 36.2 to 44.5 mg/dL, and MAPE from 27.6 % to 33.9 %. The R2 values ranged from 79.2 % to 92.1 %.

**Table 3:** Results of evaluation analysis for glucose quantification models created in the NIR-MIR region.

Method	Model	Filter	MAE (mg/dL)	RMSE (mg/dL)	MAPE (%)	R^2^ (%)
Linear	PLSR	None	25.4	35.4	28.8	93.1
		CMA	25.2	36.1	28.2	93.2
		SG	26.2	36.3	29.2	92.4
		MSC	28.3	39.4	32.8	89.3
	PCR	None	26.2	36.1	29.2	91.3
		CMA	26.3	36.3	29.3	91.4
		SG	26.0	36.2	29.1	91.5
		MSC	25.2	37.2	29.6	92.4
Nonlinear	SVR	None	25.1	34.9	28.2	93.8
		CMA	25.4	35.4	28.4	92.7
		SG	25.5	35.3	28.5	93.0
		MSC	25.2	34.8	28.0	93.6
	RFR	None	25.4	36.2	27.6	92.1
		CMA	32.6	43.5	33.9	79.8
		SG	33.2	44.5	32.3	79.2
		MSC	32.0	41.0	32.9	81.3
ANN	MLP	None	25.3	35.5	27.2	92.6
		CMA	24.0	34.7	26.5	92.8
		SG	24.4	34.0	26.9	92.2
		MSC	27.7	39.9	29.5	87.9
	LSTM	None	24.4	35.9	26.8	93.3
		CMA	24.2	36.6	27.0	92.1
		SG	24.2	36.5	26.1	92.5
		MSC	23.5	36.4	27.3	92.9

Note. ANN: Artificial Neural Networks; LSTM: Long Short-Term Memory; CMA: Convolutional Moving Average; MAE: Mean Absolute Error; MAPE: Mean Absolute Percentage Error; MSC: Multiplicative scatter Correction; NIR-MIR: Near-infrared and Mid-infrared; PCR: Principal Component Regression; PLSR: Partial Least Square Regression; R^2^: Coefficient of Determination; RMSE: Root Mean Square Error; RFR: Random Forest Regression; SG: Savitzky-Golay; SVR: Support Vector Regression.

**Fig. 5:**
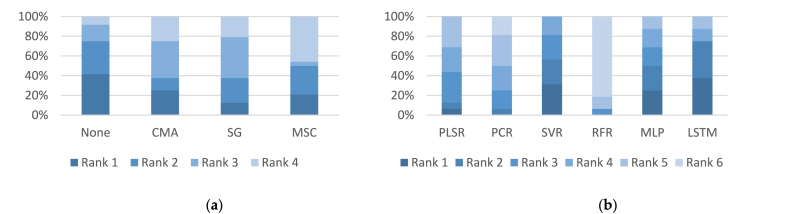
Results of comparative analysis in the NIR-MIR region between different: (**a**) filtering models; (**b**) regression models. Note. ANN: Artificial Neural Networks; LSTM: Long Short-Term Memory; CMA: Convolutional Moving Average; MSC: Multivariate Scatter Correction; MLP: Multilayer Perceptron; NIR-MIR: Near-infrared and Mid-infrared; PCR: Principal Component Regression; PLSR: Partial Least Square Regression; RFR: Random Forest Regression; SG: Savitzky-Golay; SVR: Support Vector Regression.

Moreover, Fig. 5 provides a visual comparison of model performances, illustrating that while some filtering methods like None and MSC excel, advanced regression techniques such as LSTM, SVR, and MLP show promise across varying conditions, reinforcing the importance of choosing the right combination of techniques for specific applications.

## 5 Discussion

### 5.1 NIR region

The results indicate that linear models like PLSR and PCR retain a strong predictive ability in NIR spectroscopy for glucose monitoring, potentially due to their robustness against overfitting and their efficiency in handling linear relationships. This suggests that when the underlying relationship between spectral features and glucose concentrations is primarily linear, these models are more likely to succeed.

Conversely, the poorer performance of nonlinear models such as SVR and RFR in this study might stem from several factors. Nonlinear models are typically more sensitive to the structure and nature of the data, requiring more nuanced tuning and training to capture complex patterns effectively. This sensitivity might have contributed to their underperformance, highlighting the need for further optimisation and study, especially in scenarios involving significant nonlinearity.

The similarity in performance between ANN models and linear models suggests that ANNs, despite their complexity and capability to model nonlinear relationships, might not provide additional benefits over simpler models in cases where the data relationships are not profoundly nonlinear.

The significant impact of filter choice on the models' performance underscores the critical role of pre-processing in NIR spectroscopy. Filters like CMA and SG, which effectively smooth and enhance spectral data, can significantly improve model accuracy by reducing noise and highlighting relevant spectral features. In contrast, the None filter might allow too much noise, whereas MSC, known for its ability to correct baseline shifts and scattering effects, might alter the data in ways that do not always benefit all types of models.

Given these insights, selecting the appropriate filter is paramount and should be tailored to the specific characteristics of the data and the modelling techniques used. Further research should explore the integration of advanced filtering techniques with robust modelling approaches to enhance predictive accuracy in glucose monitoring.

In the analysis of the NIR spectra, we acknowledge the presence of significant noise, as illustrated in Fig. 1 (a), which may have compromised the predictive accuracy of our models in the NIR region. This challenge is compounded by the limitations of the infrared spectrometer used, which is primarily optimised for MIR data collection and offers a relatively narrow band in the NIR spectrum. Such spectral limitations can hinder the capture of comprehensive chemical information, thereby affecting the results. Despite these constraints, the NIR region was included in our study to ensure comprehensiveness in our spectral analysis and to serve as a baseline for comparison, which is common practice in machine learning applications. This approach allows for a more holistic understanding of the spectral data, providing essential baseline information that can be critical when assessing the added value of incorporating MIR data.

### 5.2 MIR region

The results highlight that linear models, PLSR and PCR, generally maintain better performance in the MIR region compared to nonlinear models, SVR and RFR, which is consistent with the findings in the NIR region. This could indicate that linear relationships in the spectral data are more predominant, or that these models are less sensitive to the noise and variability in the MIR spectral data.

The poorer performance of the nonlinear models in this study might reflect challenges in model tuning or the intrinsic complexity of capturing nonlinear interactions in the MIR spectra. This suggests that while nonlinear models are theoretically well-suited for complex relationships, practical implementations might require more refined strategies, possibly involving more advanced preprocessing or feature extraction techniques.

The moderate performance of the ANN models, similar to that of the linear models, implies that for the MIR region, advanced modelling techniques like MLP and LSTM do not significantly outperform simpler linear approaches under the conditions tested. This could be due to the inherent noise and variability in MIR spectroscopy data, which might obscure the subtle nonlinear patterns that ANNs are capable of capturing.

The significant impact of filter choice on model performance also underscores the critical role of spectral preprocessing in achieving optimal predictive accuracy. The SG and CMA filters notably enhanced model performance across the board, suggesting their effectiveness in reducing noise and improving the signal quality of the MIR spectra.

In conclusion, this analysis emphasises the necessity of selecting appropriate machine learning models and preprocessing techniques based on the specific characteristics of the spectral region and the analytical goals. Further research is needed to investigate the integration of these models and filters in other spectral analysis settings and to refine these techniques for improved performance in glucose monitoring applications. The insights gained here are crucial for the development of accurate and reliable glucose monitoring devices and may inform future advancements in spectroscopic analysis technology.

### 5.3 NIR-MIR region

The superior performance of linear models, PLSR and PCR, in the NIR-MIR region, underscores the efficacy of linear regression techniques in handling spectral data where the relationships between variables are predominantly linear. The consistent results across different filters, especially the optimal performance with the CMA filter, suggest that linear models can robustly capture the relevant features necessary for accurate glucose prediction.

In contrast, the nonlinear models, particularly RFR, showed variability in performance, which might be attributed to their sensitivity to the non-linear complexities in the NIR-MIR spectra. However, SVR's competitive performance close to that of linear models indicates its potential when properly tuned and when used with the CMA filter, which seems to enhance model responsiveness to non-linear patterns.

The comparable performance of the ANN models to the linear ones, especially under CMA filtering, highlights ANNs' ability to model both linear and non-linear relationships effectively. However, the slight variability in their performance metrics across different filters suggests a susceptibility to preprocessing techniques, which could affect their generalizability.

The notably poorer performance of the RFR algorithm in certain configurations emphasises the challenge of parameter selection and model tuning in nonlinear regression, particularly in complex spectral analysis tasks like glucose monitoring. The significant range in performance metrics across different filters, CMA and MSC, points to the critical influence of data preprocessing on the effectiveness of nonlinear models.

Further research should focus on optimising these models, particularly exploring how different preprocessing techniques such as filtering affect their performance. Continued exploration into more advanced filtering techniques and their integration with both linear and nonlinear modelling approaches may yield improvements in accuracy and reliability for glucose monitoring applications.

The rationale for combining NIR and MIR spectroscopy in our study was driven by the intention to broaden the analysis over a wider spectral wavelength range. This strategic integration not only enhances the comprehensiveness and robustness of the predictive models but also provides a broader chemical fingerprint, potentially improving the accuracy of the analysis beyond what could be achieved by either region alone. While the NIR region alone did not yield promising results, the combination with MIR demonstrated superior performance in some scenarios compared to MIR alone, and consistently outperformed the NIR analyses. However, this superiority was not uniformly observed across all scenarios. This mixed outcome underscores the potential of the combined approach and justifies further exploration with more sophisticated spectral acquisition and data integration methods.

## 6 Conclusion

In sum, this paper focuses on the non-invasive monitoring of glucose levels and evaluates the efficacy of different machine learning techniques applied to NIR, MIR and a combined NIR-MIR spectroscopy, utilising various preprocessing filtering techniques. The results indicate that linear models, such as PLSR and PCR, exhibit superior predictive ability compared to nonlinear models, including SVR and RFR, in all three regions. The ANN models, MLP and LSTM, also demonstrated moderate predictive ability, comparable to that of linear models. Furthermore, our analysis revealed that filtering methods can greatly enhance the accuracy of the predictive models. The CMA and SG filters were found to be the most effective in terms of accuracy, producing lower MAE, RMSE, and MAPE values, and higher R^2^ values across all models tested. On the other hand, no filtering may be more suitable for certain prediction models. Our study emphasises the importance of selecting the appropriate filter for glucose quantification using NIR and MIR spectroscopy. Moreover, the results suggest that the performance of the different models varied depending on the algorithm and filter used. Furthermore, our findings indicate that the combination of NIR and MIR spectra does not inherently lead to improved analysis, emphasising the need for the implementation of effective and advanced data fusion techniques.

## 7 Future work

In order to build upon the findings of this research, future work may involve:

• Exploring a broader spectrum of preprocessing filtering techniques and machine learning algorithms, which could further optimise methods for glucose monitoring.
• Investigating different methods beyond the traditional absorbance Fourier transform to optimise signal-to-noise ratio in NIR readings.
• Exploring how various variable selection techniques may influence the precision of glucose quantification analysis. Such a comparison might yield additional insights into the model's performance.
• Investigating a wider NIR waveband or other spectral regions, including terahertz and Raman spectroscopy. This could contribute significantly to the development of new and improved glucose quantification technologies.
• Examining the effects of combining different pre-processing filters, a strategy that might enhance calibration accuracy.
• Integrating multiple sensing modalities, such as optical, electrical, and thermal sensing. This could provide a more comprehensive and accurate approach to glucose monitoring.
• Employing a dedicated NIR spectrometer to improve data quality and analytical performance.

This approach lays out a variety of potential research avenues that could provide valuable insights and advancements in the field of glucose monitoring.

## Funding

This research received no external funding.

## Data availability statement

For the analysis, we coded in Python (3.6.7) [76]. The libraries used include; Pandas [77], NumPy [78], and Sklearn [79]. Our implementation codes are publicly accessible on this Gitlab repository.

## CRediT authorship contribution statement

**Heydar Khadem:** Writing – review & editing, Writing – original draft, Visualization, Validation, Software, Methodology, Investigation, Formal analysis, Data curation, Conceptualization. **Hoda Nemat:** Writing – review & editing, Methodology, Conceptualization. **Jackie Elliott:** Writing – review & editing, Supervision. **Mohammed Benaissa:** Writing – review & editing, Supervision, Resources, Conceptualization.

## Declaration of competing interest

The authors declare that they have no known competing financial interests or personal relationships that could have appeared to influence the work reported in this paper.

## AbbreviationsList

ANN: Artificial Neural Networks
DCW-PAS: Differential Continuous Wave Photoacoustic Spectroscopy
LSTM: Long Short-Term Memory
CMA: Convolutional Moving Average
MAE: Mean Absolute Error
MIR: Mid-infrared
MLP: Multilayer Perceptron
MSC: Multiplicative scatter Correction
NIR: Near-infrared
NIR-MIR: Near-infrared and Mid-infrared
PCR: Principal Component Regression
PLSR: Partial Least Square Regression
R2: Coefficient of Determination
RFR: Random Forest Regression
RMSE: Root Mean Square Error
SG: Savitz-ky-Golay
SVR: Support Vector Regression
